# Investigation of the factors influencing spinal manipulative therapy force transmission through the thorax: a cadaveric study

**DOI:** 10.1186/s12998-023-00493-1

**Published:** 2023-08-07

**Authors:** Jérémie Mikhail, Martha Funabashi, Stéphane Sobczak, Martin Descarreaux, Isabelle Pagé

**Affiliations:** 1 Department of Chiropractic, Université du Québec à Trois-Rivières, 3351 Boul. des Forges, Trois-Rivières, QC G8Z 4M3 Canada; 2 Research Group on Neuromusculoskeletal Disorders, Université du Québec à Trois-Rivières, 3351 Boul. des Forges, Trois-Rivières, QC G8Z 4M3 Canada; 3 Canadian Memorial Chiropractic College, 6100 Leslie St, North York, ON M2H 3J1 Canada; 4 Department of Anatomy, Université du Québec à Trois-Rivières, 3351 Boul. des Forges, Trois-Rivières, QC G8Z 4M3 Canada; 5 Department of Human Kinetics, Université du Québec à Trois-Rivières, 3351 Boul. des Forges, Trois-Rivières, QC G8Z 4M3 Canada; 6 Center for Interdisciplinary Research in Rehabilitation and Social Integration (Cirris), Centre Intégré Universitaire de Santé et de Services Sociaux de la Capitale-Nationale (CIUSSS-CN), 525 Boul. Wilfrid-Hamel, Québec City, QC G1M 2S8 Canada; 7 Research Chair in Functional Anatomy, Université du Québec à Trois-Rivières, 3351 Boul. des Forges, QC G8Z 4M3 Trois-Rivières, Canada

**Keywords:** Spinal manipulation, Spinal mobilization, Force–time characteristics, Patient-table interface, Clinician-patient interface, Cadaveric study, Kinetic, Kinematic

## Abstract

**Background:**

Spinal manipulative therapy (SMT) clinical effects are believed to be linked to its force–time profile characteristics. Previous studies have revealed that the force measured at the patient-table interface is most commonly greater than the one applied at the clinician-patient interface. The factors explaining this force amplification remains unclear.

**Objective:**

To determine the difference between the force applied to a cadaveric specimen’s thoracic spine and the resulting force measured by a force-sensing table, as well as to evaluate the relationship between this difference and both the SMT force–time characteristics and the specimens’ characteristics.

**Methods:**

Twenty-five SMTs with different force–time profiles were delivered by an apparatus at the T7 vertebra of nine human cadaveric specimens lying prone on a treatment table equipped with a force plate. The difference between the force applied by the apparatus and the resulting force measured by the force plate was calculated in absolute force (F_diff_) and as the percentage of the applied force (F_diff%_). Kinematics markers were inserted into T6 to T8 spinous and transverse processes to evaluate vertebral displacements during the SMT thrusts. Mixed-effects linear models were run to evaluate the variance in F_diff_ and F_diff%_ explained by SMT characteristics (peak force, thrust duration and force application rate), T6 to T8 relative and total displacements, and specimens’ characteristics (BMI, height, weight, kyphosis angle, thoracic thickness).

**Results:**

Sixty percent of the trials showed lower force measured at the force plate than the one applied at T7. F_diff_¸ was significantly predicted (R^2^_marginal_ = 0.54) by peak force, thrust duration, thoracic thickness and T6–T7 relative displacement in the z-axis (postero-anterior). F_diff%_ was significantly predicted (R^2^_marginal_ = 0.56) by force application rate, thoracic thickness and total T6 displacements. For both dependant variables, thoracic thickness showed the highest R^2^_marginal_ out of all predictors.

**Conclusion:**

Difference in force between the clinician-patient and the patient-table interfaces is influenced by SMT force–time characteristics and by thoracic thickness. How these differences in force are associated with vertebral displacements remains unclear. Although further studies are needed, clinicians should consider thorax thickness as a possible modulator of forces being transmitted through it during prone SMT procedures.

**Supplementary Information:**

The online version contains supplementary material available at 10.1186/s12998-023-00493-1.

## Background

Spinal manipulative therapy (SMT) is a conservative intervention commonly used by chiropractors in the management of musculoskeletal conditions [1]; it is believed to trigger neuromechanical responses that potentially contribute to its therapeutic effects [2]. From a clinical standpoint, the application of SMT involves modulating the force–time characteristics of the applied force to accommodate each patient’s unique clinical and individual characteristics [3]. Therefore, quantifying all forces acting on the body during the application of SMT can contribute to advancing our understanding of SMT mechanisms.

In previous studies, the forces applied during SMT at the thoracic spine have been extensively quantified. However, most of these studies have characterized the forces measured at either the clinician-patient or patient-table interface, as reported in a systematic review by Downie et al. which focused on SMT performed by clinicians [4]. The authors reported that the SMT forces measured at the clinician-patient interface range from 238 to 561 N [5–7]. It should be noted that this range of forces does not include a reported standard deviation. Meanwhile, peak thrust forces assessed at the patient-table interface averaged 1044 N (± 186 N). The concurrent measurement of SMT forces at both the clinician-patient and patient-table interfaces has been investigated in a few studies [8–11]. Kirstukas & Backman [8] observed that peak forces of SMT delivered by clinicians at the patient-table interface were, on average, 16% lower than the peak forces delivered at the clinician-patient interface. Recent studies using more advanced force-sensing technologies, reported that SMT forces delivered by an apparatus or a clinician were about 14% higher at the patient-table interface than the ones at the clinician-patient interface [9, 10]. Higher forces at the patient-table interface are consistent with the impact mechanics of deformable bodies [12, 13]. According to Thomas et al. [10], the ballistic nature of the high-velocity dynamic characteristic of SMT forces may not provide sufficient time for muscle moments to counteract the SMT. Consequently, forces at the participant–table interface probably represent the interaction between external applied forces, gravito-inertial forces (including thorax’s mass), and relationships between the segments of the spine and other adjacent body segments.

Interestingly, Funabashi et al. [11] measured SMT forces applied simultaneously to older adults at the clinician-patient and patient-table interfaces, and observed greater forces at the clinician-patient interface in most participants. The authors speculated that the degenerative changes in older adults’ thorax may have influenced the forces acting on internal tissues during SMT. Indeed, Mikhail et al. [9] observed that not only SMT force–time characteristics, but also participant characteristics, such as sex and thoracic thickness, could influence the difference between the forces measured at the clinician-patient and patient-table interfaces. This highlights the importance of understanding how different interventions and patients’ characteristics influence SMT forces’ impact on the body. Identifying the factors that influence the difference between forces applied by a clinician during SMT and the forces experienced by a patient on the treatment table can lead to significant advancements in elucidating SMT’s biomechanics and underlying mechanisms. This understanding could inform future studies using devices and technologies to measure the SMT force–time characteristics, emphasizing the need for standardization and careful analysis of relevant factors.

Therefore, the primary objective of the present study was to explore, using cadaveric models, the role of the SMT force–time characteristics and of the patients anthropometric characteristics on the difference between the force applied at the clinician-patient interface and the force measured at the patient-table interface. Based on previous studies [9, 10], it was hypothesized that the force measured at the patient-table interface would be greater than the force at the clinician-patient interface. Moreover, it was hypothesized that the force differences would be related to the specimens’ anthropometric characteristics, and that an increase in the force amplification would be observed when the SMT rate of force application increased.

## Methods

### Specimens

Nine fresh-frozen human cadavers were obtained as part of the University Willed Body Donation Program. The cadavers had not undergone any surgery or been subjected to procedures in the anatomy laboratory that could have affected the biomechanical properties of the tissues in the spine, thorax or cervical regions. This study was conducted between July and August 2021 and was approved by the Université du Québec à Trois-Rivières Ethics Committee (SCELERA-21–03).

### Protocol Summary

Each procedure is detailed below. Briefly, fresh-frozen (i.e., unembalmed) cadaveric specimens were thawed at room temperature (18 °C) for 36 h and positioned in a prone position. Specimens were then instrumented with kinematic markers at T6 to T8 vertebrae and X-rays were taken to measure the kyphosis angle. Next, specimens were transferred on a force-sensing table and the thoracic thickness were measured. An apparatus using a servo-linear motor [14] was then used to measure spinal stiffness at T7. The same apparatus was then used to apply 25 SMTs, every 2 min, progressively increasing the peak force and the rate of force application. The direction of the SMT was perpendicular to the table. Specimens were prepared and data were collected in an 18 °C room temperature with the data acquisition process ranging from 2 to 3 h.

### Kinematic instrumentation

**Fig. 1:**
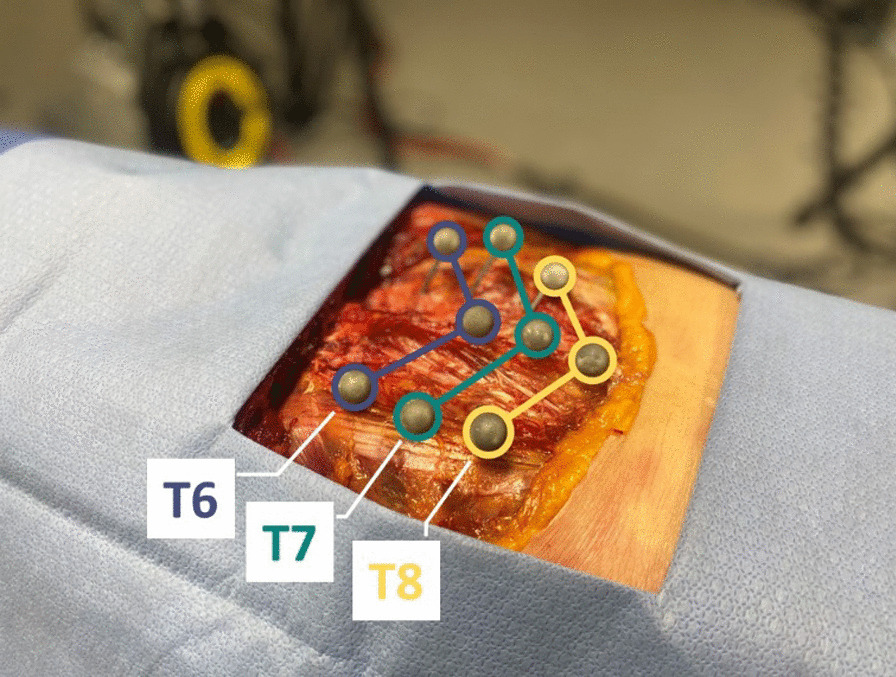
Kinematic markers placements. Three markers were inserted into T6 to T8 spinous processes and six markers into their left and right transverse processes

The skin and superficial muscle layers were removed to allow proper placement of the kinematic markers. Nine passive markers (5 mm diameter spheres) glued on metal rods (gauge = 2 mm) were drilled into the spinous process and both transverse processes of T6, T7 and T8 vertebrae, forming a kinematic rigid body for each vertebra (Fig. 1). X-rays were taken to confirm proper vertebrae identification and rod insertion.

### Anthropometric measurements

The height, weight, age, and sex (male, female) of each specimen were obtained from their respective record. Once the kinematic markers were placed, and without moving the specimens, a lateral chest X-ray from T3 to L1 was taken to measure the thoracic kyphosis angle. The centroid technique was used to calculate the regional angle adjacent to the contact at T5/T6 and T7/T8 [15]. Corners of T5 to T8 vertebral bodies were marked as reference points, excluding overt osteophytes. Lines were then drawn between opposite corners of the same vertebral body. Finally, the intersection angle of two lines, each passing through the centroid of adjacent vertebrae, was used to determine the regional thoracic kyphosis angle. Three reviewers with relevant experience in X-ray films reading evaluated the angles separately, and an intraclass correlation coefficient (ICC) was calculated to ensure reliability. The reliability was considered “good”, with an ICC of 0.89. The average of the three reviewers’ measurements was then used for further analyses.

The specimens were then transferred to the instrumented table and placed in the prone position with both hands resting above their head on the table head piece. A caliper (S&S X-ray Products Inc. Brooklyn, NY, error ± 0.5 cm) was used to measure the thoracic thickness, defined as the shortest distance between the tip of T7 spinous process and the surface of the table.

### Spinal Stiffness

The spinal stiffness of each specimen was assessed prior to the application of the SMTs. A single rounded-tip indenter was installed on the apparatus (18 mm diameter) and positioned over the T7 vertebral lamina. The apparatus then gradually applied force using a constant rate of 18 N/s, starting with 5 N, up to a peak force of 45 N maintained for 1 s. This method has been shown to be reliable for assessing thoracic spinal stiffness [16]. The measure was repeated five times, separated by at least one minute to allow the tissues to return to a rest state. The apparatus indenter displacement (mm) was recorded during each trial.

### SMT Application

The single tip indenter was replaced by a double-tip indenter (θ tip = 10 mm; distance between the centre of the tips = 56 mm). For standardization, the indenter tips were positioned medially to the kinematic markers at T7 transverse processes.

**Fig. 2:**
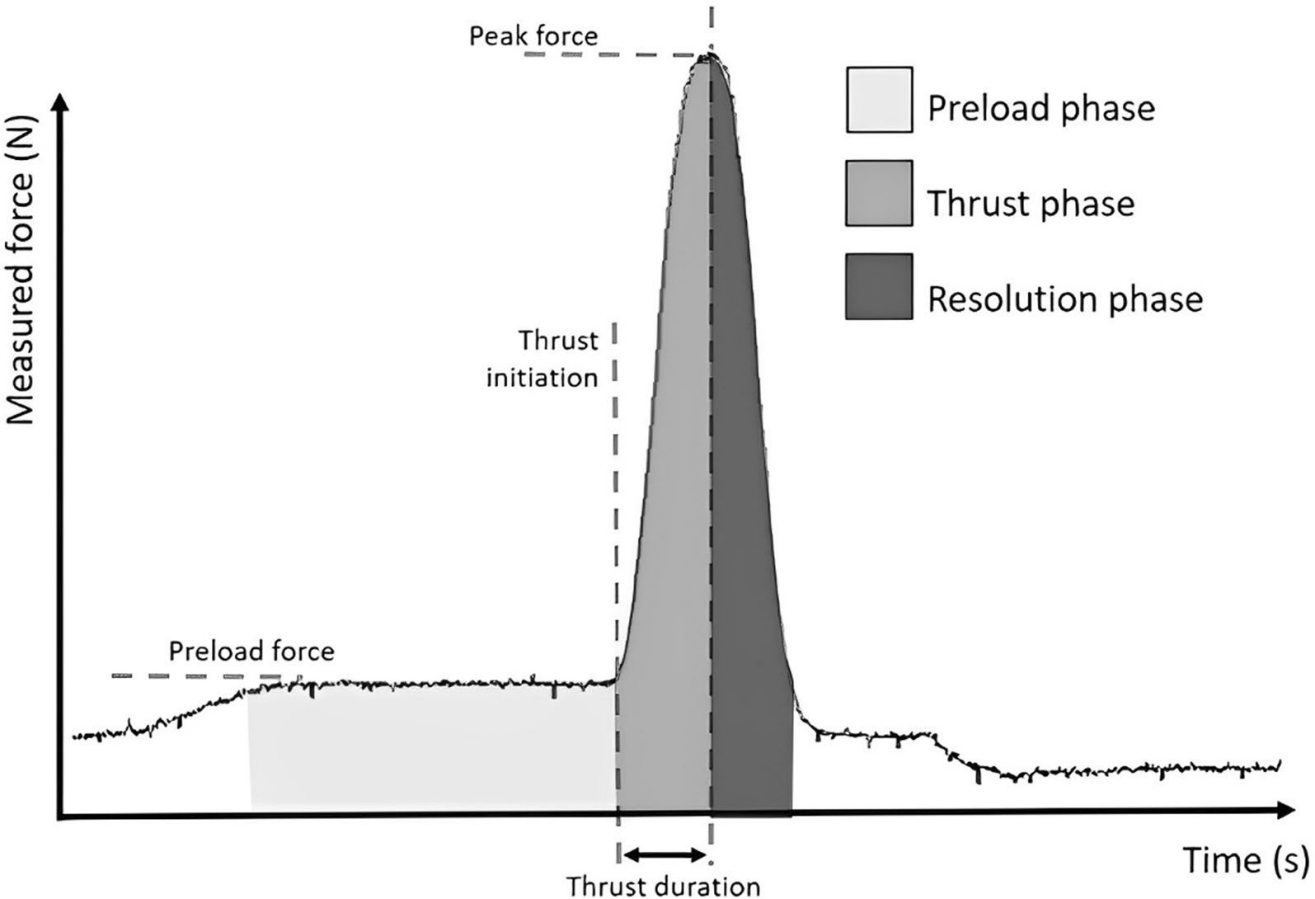
Typical SMT force–time curve and the biomechanical parameters. The rate of force application is calculated by dividing the difference between the peak force and preload force by the thrust duration

Typical SMT force–time graphs are shown in Fig. 2. A total of 25 SMTs, 2 min apart, were delivered by the apparatus to each specimen at T7 level (Table 1). A constant preload of 20 N was used across SMTs. The initial peak force was set at 200 N, and increased in 100 N increment to reach a maximum peak force of 600 N. Each peak force was delivered using an increasing rate of force application ranging from 500 N/s to 2500 N/s, in 500 N/s increments. The force–time characteristics used in this study were based on the ones reported in the literature [17–19]. The lowest peak force was first delivered with the five different rates of force application, then the second-lowest peak force was delivered, and so on until the highest peak force was delivered with the highest rate of force application. This sequence was adopted to progressively load the specimen and thus minimize the impact of changes in tissues generated by higher peak forces. The displacement of the apparatus indenter (mm) was recorded during each trial, along with the forces exerted by the force-sensing table and the spine kinematics.

**Table 1:** SMT force–time characteristics applied to the cadaveric specimens

	SMT Peak force (N)*	Thrust duration (s)	SMT rate of force (N/s)
SMT1	200	0.360	500
SMT2	200	0.180	1000
SMT3	200	0.120	1500
SMT4	200	0.090	2000
SMT5	200	0.072	2500
SMT6	300	0.560	500
SMT7	300	0.280	1000
SMT8	300	0.187	1500
SMT9	300	0.140	2000
SMT10	300	0.112	2500
SMT11	400	0.760	500
SMT12	400	0.380	1000
SMT13	400	0.253	1500
SMT14	400	0.190	2000
SMT15	400	0.152	2500
SMT16	500	0.960	500
SMT17	500	0.480	1000
SMT18	500	0.320	1500
SMT19	500	0.240	2000
SMT20	500	0.192	2500
SMT21	600	1.160	500
SMT22	600	0.580	1000
SMT23	600	0.387	1500
SMT24	600	0.290	2000
SMT25	600	0.232	2500

^*^ The peak force includes the preload force of 20 N

### Apparatus used to deliver SMTs and measure spinal stiffness

The apparatus for measuring spinal stiffness and delivering the SMTs used a servocontrolled linear actuator motor (Linear Motor Series P01–48 × 360, LinMot Inc., Zurich, Switzerland). This apparatus has been shown to deliver a target force with high repeatability and precision [14].

### Force-sensing table

A treatment table with an integrated force plate was used to assess the forces at the anterior portion of the thorax during each SMT. Specifically, the Force Sensing Table Technology (FSTT®, Canadian Memorial Chiropractic College, Toronto, ON, Canada) consists of a treatment table and an embedded AMTI force plate (Advanced Mechanical Technology Inc., Watertown, 50 × 50 cm, Massachusetts, USA). The FSTT® is reliable in the measurement of SMT force–time characteristics [20]. The cushion that is normally on the load platform was removed to avoid the addition of a deformable material between the specimens and the table. Proper calibration procedures were applied to both the force-applying apparatus and the force-sensing table prior to the experimentation.

### Kinematic measurement

Eight cameras (PrimeX22, Optitrack, NaturalPoint Inc., Corvallis, OR, USA) with positional error resolution of less than ± 0.15 mm and rotational error resolution of less than 0.5 degrees were positioned around the experimentation area. The three-dimensional kinematic was assessed, forming rigid bodies of three kinematic markers inserted in the same vertebrae. Kinematics data, together with the load platform, indenter displacement and applied force data were all recorded at 360 Hz using *Motive (Optitrack,* NaturalPoint Inc., Corvallis, OR, USA) software.

### Data acquisition synchronization

The equipment was synchronized with multiple pre-testing trials. Kinematic markers were placed on the apparatus’ indenter and performed SMT on the force plate using the same profile as our experiment. Simultaneously, a multi-channel data acquisition system recorded force and motion data. The data was processed to ensure accurate and reliable measurements.

### Data processing and analysis

#### Data filtering

The apparatus, force plate and the kinematic data were filtered using a smoothing window of 3 (0.002778 s per window, or 360 Hz).

#### Spinal stiffness calculation

A MATLAB (MathWorks, Natick, Massachusetts, USA) script was developed to calculate both the global and terminal spinal stiffness coefficients using the force and displacement data from each spinal stiffness measurement. The first two measurements were discarded to allow tissue preconditioning, and the average of the last three measurements was used for further analysis. Global stiffness was defined as the slope of the straight line best fitting the force–displacement data between 10 and 45 N, while terminal stiffness was defined as the ratio of the variation in force and displacement between 10 and 45 N [16].

#### Force-sensing table and apparatus data processing

**Fig. 3:**
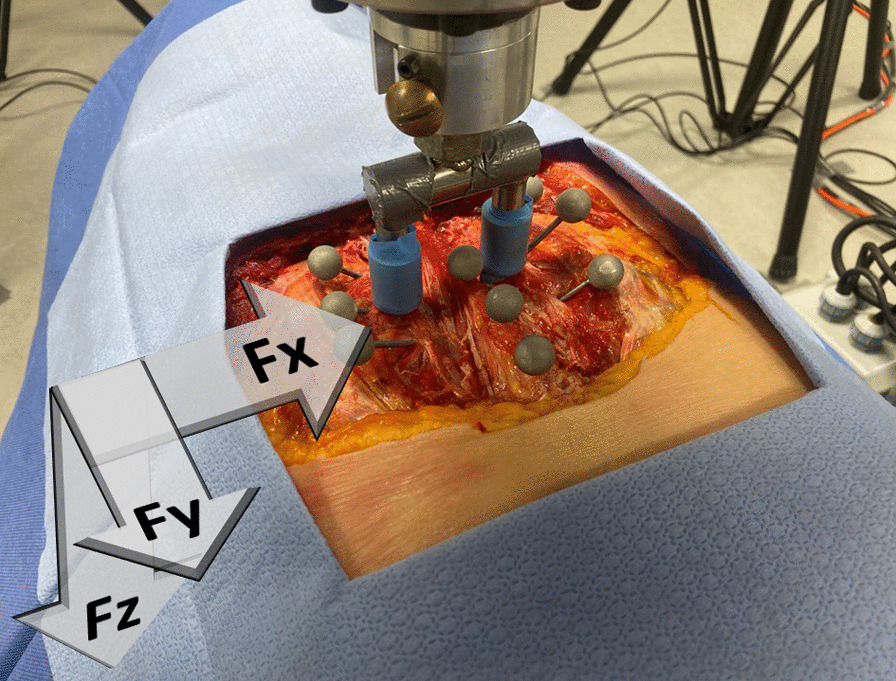
Visualization of the coordinate system used for the force-sensing table measurements. The same coordinate system was used for the kinematics data

The data from the apparatus and from the force-sensing table were processed in parallel. Data from each trial were imported and processed with *R Studio (*version 2022.02.3 + 492, Posit, PBC). Data from the apparatus and force-sensing table were first converted into force values using the coefficient determined during the calibration procedures. As the force-sensing table measures forces in the three axes of motion (Fig. 3), the resultant force was used for analyses: Fr = $\sqrt{{\mathrm{Fx}}^{2}+ {\mathrm{Fy}}^{2}+ {\mathrm{Fz}}^{2}}$. Data were imported into an existing LabView (National Instruments, Austin, Texas) script to automatically identify the peak force, while the thrust initiation was manually marked by visually inspecting the force–time curve. The marking process entailed identifying data points in the z-axis, which were then exported to the x-axis and y-axis. The primary author, who had experience with this methodology, performed all the markings. Thrust duration, rate of force application, and apparatus indenter displacement (total and as the percentage of the thoracic thickness) were also calculated.

#### Kinematics data processing

First, the displacement of each vertebra (T6 to T8) in the z-axis during the SMT thrust phase was calculated. Each vertebra total displacement ($\Delta \mathrm{s}$) was also calculated using a procedure similar to the one used for the force-sensing table data: $\Delta \mathrm{s}=$ $\sqrt{{\Delta \mathrm{sFx}}^{2}+ \Delta {\mathrm{sFy}}^{2}+ \Delta {\mathrm{sFz}}^{2}}$. The relative displacement between T6–T7 and T7–T8 was also calculated by taking into account the z-axis displacement and the total displacement.

#### Calculation of the difference between the applied and the measured forces

The difference between the peak force applied by the apparatus (F_applied_) and the peak force measured by the force-sensing table (F_table_) was calculated for each trial using the method proposed by Mikhail et al. [9]. The force difference (F_diff_) was then determined by subtracting F_applied_ from F_table_. A positive F_diff_ therefore corresponds to a greater force measured by the force-sensing table than the force applied. To facilitate comparison between peak forces, F_diff_ was also expressed as a percentage of the applied peak force: ${F}_{diff\%}= \frac{{F}_{diff}}{{F}_{applied}} \times 100$.

### Statistical analyses

#### Descriptive analysis.

All specimen characteristics (i.e., age, sex, height, weight, chest thickness, BMI, kyphosis angle, global and terminal stiffness) were considered to be non-normally distributed due to the number of specimens (n = 9). The normality of the other variables (F_diff_, F_diff%_, indenter displacements and kinematic variables) was assessed using visual inspection of the data distribution using histogram graphs, as well as Skewness and Kurtosis. Specimen characteristics, indenter displacement from thrust initiation to peak force and kinematics data were first described using mean and standard deviation (SD) for parametric data and median and inter-quartile range for nonparametric data. For each SMT force–time profile, the mean (with SD) of F_diff_ and F_diff%_ were calculated as well as the percentage of trials resulting in a higher peak force at the force-sensing table interface.

#### Main analysis

Mixed-effects models were used to analyze possible correlations between the difference between the applied force and the force measured by the force-sensing table (F_diff_ and F_diff%_), the SMT force–time characteristics and the specimens’ characteristics [21]. This approach accounts for the nested nature of a range of modalities performed on a group of different subjects with their own explanatory variables. A multilevel regression model for two-level data was used, with the SMT characteristics as level 1 and the specimens’ characteristics as level 2. Another advantage of mixed effects models is their capacity to handle missing data.

Level 1 variables (peak force; thrust duration; rate of force application) and level 2 variables (body mass index [BMI]; height; weight; chest thickness; global and terminal spinal stiffness; T6, T7 and T8 total displacements; T5–T6 and T6–T7 relative displacements; total indenter displacement; indenter displacement as a percentage of the thoracic thickness) were set as fixed effects. They are expected to operate in a predictable way across a range of participants and conditions. On the opposite, the specimens were set as random effects as clusters of dependent data, where they could have behaved differently from the average trend. *R studio* with the *lme4* package were used for the analyses [22].

Mixed-effects models were independently computed for the two dependent variables (F_diff_ and F_diff%_). First, level one variables were entered in mixed-effects models. Significantly correlated variables (*p* < 0.05) were kept for the second step. If different variables showed similar significance (i.e., similar *p* values), the one with the highest marginal R^2^ was kept. When the model with the best fitting SMT characteristics was found, level 2 variables were introduced.

To determine which specimen characteristics to enter in the final model, each characteristic was first independently introduced within the level 1 based model to identify significant relationships. The procedure used for level 1 variables was also used to determine which specimen characteristics to combine in the final model (i.e., *p* value and marginal R^2^). Moreover, if multiple variables measuring related concepts (e.g., vertebra displacement in the z-axis and total movement of the same vertebra) were significant, two pre-final models were computed and compared to identify the most relevant one. Finally, if a variable lost significant correlation when combined with other variables, it was discarded in a refined model.

## Results

### Specimens

Table 2 presents the specimen characteristics. A total of 9 specimens (6 females: 3 males) aged between 70 and 87 years old were used for the current study.

**Table 2:** Specimen characteristics

Characteristics	Value
Females: Males	6:3
Age (years; median [IQR])	82.00 [8.00]
Height (m; median [IQR])	1.57 [0.08]
Weight (kg; median [IQR])	54.40 [11.30]
BMI (kg/m^2^; median [IQR])	22.00 [2.00]
Chest thickness (mm; median [IQR])	175.00 [35.00]
Thoracic kyphosis angle (°; median [IQR])	16.60 [3.79]
Global spinal stiffness (N/mm; median [IQR])	12.64 [5.65]
Terminal spinal stiffness (N/mm; median [IQR])	12.83 [5.95]

### Indenter displacement and kinematics during SMT.

The indenter displacement as well as absolute and relative vertebral displacements from thrust initiation to peak force are presented in Additionnal file 1. The total indenter displacements ranged from 6.20 to 36.98 mm, depending on the SMT force–time profile (mean = 18.89 mm, SD = 7.71 mm). When reported as a percentage of the thoracic thickness, the results ranged from 3.07 to 23.11% (median = 9.99%, IQR = 7.29%). Full kinematic data were available for 217 of the 225 trials (loss of 3.56% of the trials). T6 showed the highest mean of absolute displacement for both the Z-axis (mean = 12.55 mm, SD = 5.83 mm) and the total movement (mean = 13.00, SD = 6.05 mm). T7 followed in both variables (Z-axis, mean = 11.99 mm, SD = 5.71 mm; total, mean = 12.68, SD = 5.71 mm) and T8 demonstrated the lowest mean of absolute displacement (Z-axis, mean = 11.31 mm, SD = 5.27 mm; total, mean = 12.11, SD = 5.53 mm). In terms of the relative displacement, T6/T7 measured an average of movement 0.16 mm (SD = 1.03 mm) in the Z-axis and a mean of 1.52 mm (SD = 0.078 mm), compared to T7/T8 with a higher displacement in the Z-axis (mean = 1.03, SD = 0.82), but a lower one in total displacement (mean = 1.53 mm, SD = 0.78 mm).

### Difference between the applied and measured forces

The mean, standard deviation, and range of the F_diff_ and F_diff%_ are reported in Table 3 for each applied SMT. In 60% of the trials, forces measured at the patient-table interface were lower than the force at the clinician-patient interface. No force data were corrupted or lost during the study. The difference in force ranged from an increase of 76.6 newtons (N) to a decrease of 114.3 N, with an interquartile range of 48.6 N. Mean value of F_diff_ and F_diff%_ calculated for each of the 9 specimens in function of the SMT force–time profile can be seen in Additional file 2: 1A and B.

**Table 3:** Differences between the peak force applied by the apparatus and the one measured by the force-sensing table for the 25 SMTs

	F_diff_ (N)*		F_diff%_ (%)		Number of cadavers showing higher force measured by the force-sensing table than the applied force
	Mean (SD)	Range	Mean (SD)	Range	
SMT1	− 15.78 (18.11)	− 41.77 to 18.73	− 7.8 (9.0)	− 20.7 to 9.3	2/9
SMT2	− 14.64 (17.83)	− 39.31 to 20.17	− 7.4 (9.0)	− 19.8 to 10.3	2/9
SMT3	− 1.85 (20.98)	− 26.45 to 45.04	− 0.9 (11.0)	− 13.8 to 23.8	4/9
SMT4	14.92 (22.77)	− 11.24 to 66.42	7.9 (12.1)	− 6.0 to 35.4	7/9
SMT5	23.66 (24.86)	− 6.61 to 76.65	12.4 (13.1)	− 3.4 to 40.2	7/9
SMT6	− 17.36 (30.96)	− 63.69 to 29.51	− 5.7 (10.2)	− 21.0 to 9.8	3/9
SMT7	− 18.01 (30.20)	− 62.45 to 31.18	− 6.0 (10.1)	− 20.8 to 10.5	3/9
SMT8	− 16.50 (29.85)	− 59.29 to 32.52	− 5.6 (10.2)	− 20.2 to 11.1	3/9
SMT9	− 7.81 (29.93)	− 47.63 to 44.43	− 2.7 (10.4)	− 16.5 to 15.5	4/9
SMT10	12.81 (24.49)	− 23.96 to 46.28	4.5 (8.7)	− 8.5 to 16.2	6/9
SMT11	− 21.45 (39.25)	− 81.06 to 32.38	− 5.3 (9.8)	− 20.1 to 8.1	4/9
SMT12	− 20.70 (39.59)	− 79.87 to 33.62	− 5.2 (9.9)	− 20.0 to 8.4	3/9
SMT13	− 20.12 (39.47)	− 78.88 to 35.29	− 5.1 (10.0)	− 20.0 to 9.0	3/9
SMT14	− 18.04 (38.20)	− 76.81 to 36.88	− 4.6 (9.8)	− 19.7 to 9.5	4/9
SMT15	− 11.66 (44.94)	− 74.64 to 49.35	− 3.0 (11.7)	− 19.4 12.9	4/9
SMT16	− 14.55 (46.05)	− 95.07 to 61.55	− 2.9 (9.2)	− 19.0 to 12.1	3/9
SMT17	− 17.71 (37.51)	− 92.96 to 30.53	− 3.5 (7.5)	− 18.6 to 6.1	3/9
SMT18	− 16.85 (36.38)	− 90.22 to 29.26	− 3.4 (7.3)	− 18.2 to 5.9	3/9
SMT19	− 16.11 (36.25)	− 91.18 to 28.81	− 3.3 (7.4)	− 18.5 to 5.9	3/9
SMT20	− 11.29 (35.57)	− 86.48 to 27.10	− 2.3 (7.3)	− 17.7 to 5.6	4/9
SMT21	− 21.03 (42.25)	− 110.95 to 28.80	− 3.5 (7.0)	− 18.5 to 4.8	3/9
SMT22	− 21.53 (43.18)	− 114.34 to 27.17	− 3.6 (7.2)	− 19.1 to 4.5	3/9
SMT23	− 21.42 (42.91)	− 113.32 to 29.88	− 3.6 (7.2)	− 19.0 to 5.0	3/9
SMT24	− 22.27 (42.53)	− 113.21 to 30.50	− 3.8 (7.2)	− 19.1 to 5.2	3/9
SMT25	− 21.52 (42.35)	− 113.25 to 28.14	− 3.7 (7.2)	− 19.3 to 4.8	3/9

*A positive F_diff_ means a greater force measured by the force-sensing table than the applied force

### Mixed-effects models

The final regression model for F_diff_ (marginal R^2^ = 0.54, conditional R^2^ = 0.78) and F_diff%_ (marginal R^2^ = 0.56, conditional R^2^ = 0.75) were both statistically significant and are reported in Table 4. At level 1 (i.e., SMT force–time characteristics), peak force (β = − 0.04, *p* < 0.001) and thrust duration (β = − 12.62, *p* = 0.03) were found to significantly predicted F_diff_, indicating that F_diff_ gets closer to zero with the increase in peak force and thrust duration for cadavers in which an amplification of the applied force was observed. For cadavers showing a reduction of the applied force, the results revealed a further reduction of the applied force with the increase in peak force and thrust duration. When controlled for the applied peak force (i.e., F_diff%_), the rate of force application was the only significant variable at level 1 (β = 0.004, *p* < 0.001), indicating that, regardless of peak force, an increase in the rate of force application results in a reduction in the difference of force at interfaces for the cadavers, showing a force reduction and a greater difference in the cadavers who showed force amplification.

**Table 4:** Final mixed-effects models for the difference in the applied and measured force (F_diff_) and expressed as a percentage of the applied force (F_diff%_)

	F_diff_	F_diff%_
Fixed effects	Estimates (95% confidence interval)	Estimates (95% confidence interval)
(Intercept)	− 213.46 (− 318.09 to − 108.82) **	− 64.49 (− 90.97 to − 38.02) **
SMT peak force (N)	− 0.04 (− 0.06 to − 0.02) **	–
SMT thrust duration (s)	− 12.62 (− 23.99 to − 1.25) *	–
SMT Rate of Force (N/s)	–	0.004 (0.003 to 0.005) **
Thoracic Thickness (mm)	0.12 (0.65 to 1.80) **	0.33 (0.18 to 0.47) **
T6/7 relative displacement in z− axis (mm)	− 3.96 (− 7.64 to − 0.28) *	–
T6 total displacement (mm)	–	− 0.23 (− 0.36 to − 0.09) **

Random effect	Values	Values
σ ^2^	300.57	26.24
τ _00_	330.07 _cadaver_	20.92 _cadaver_
Intraclass correlation coefficient	0.52	0.44
N	9 _cadavers_	

Model statistics	Values	Values
Observations	207	222
Marginal R^2^	0.54	0.56
Conditional R^2^	0.78	0.75

*Significant estimates at *p* < 0.05

**Significant estimates at *p* < 0.001

At level 2 (specimen characteristics, indenter displacement, and kinematics), thoracic thickness significantly predicted both F_diff_ (β = 0.12, *p* < 0.001) and F_diff%_ (β = 0.33, *p* < 0.001), indicating that thicker cadaver showed a greater amplification of the measured force, while thinner cadavers showed greater reduction in the measured force. In terms of kinematics variables, the T6–T7 relative displacement in the z-axis was the only variable to remain in the final model for F_diff_ (β = − 3.96, *p* = 0.04). For F_diff%_, both T6 and T8 total displacements were statistically significant when models were computed with one variable. Considering the collinearity between T6 and T8 total displacement variables, two final models including one of these variables were computed, and their marginal R squared were compared. The final model with T6 total displacement showed the highest marginal R squared and was therefore retained (models R^2^_marginal_ = 0.57 vs. 0.55; T6 β = − 0.23, *p* < 0.001).

For both dependant variables, thoracic thickness showed the highest marginal R^2^ when tested as the sole specimen predictor (F_diff_ R^2^ = 0.52 and F_diff%_ R^2^ = 0.55). When compared with the marginal R^2^ of the final models (i.e., 0.54 for F_diff_ and 0.56 for F_diff%_), this indicates that thoracic thickness was the main contributor for both models. The variation of F_diff_ and F_diff%_ in function of the thoracic thickness can be visualized in Additional file 3: 2A and B.

## Discussion

In this study, 25 simulated SMT with different force–time characteristics were delivered to the T7 transverse processes of 9 thawed fresh cadaveric specimens using a mechanical apparatus. Overall, the results revealed that, in 60% of the SMTs, transmitted forces were lower than the applied forces; however, some specimens presented higher transmitted forces. Our analyses indicate that the thoracic thickness may partly explain this variation between specimens, with cadavers with thicker thorax presenting higher transmitted forces, and cadavers with thinner thorax presenting lower transmitted forces, compared to the applied ones. The results further revealed that the SMT peak force, thrust duration and rate of force application, as well as vertebral kinematics, also influenced the magnitude of transmitted forces.

Several studies have investigated the effects of SMT force–time characteristics on human biomechanics or physiological responses [19], but only a handful have simultaneously measured the force applied at the clinician-patient interface and the resultant force at the patient-table interface [8–11]. While some studies observed greater forces at the clinician-patient interface (applied forces) [11], others reported greater forces at the patient-table interface (transmitted forces) [8–10]. A previous study has reported that both SMT and patient characteristics shape the behaviour and transmission of SMT forces through the human body [9]. The current study was conducted on cadaveric specimens to further investigate the exact role of SMT force–time characteristics and patient characteristics on the SMT force behaviour. Interestingly, results from this study were not completely aligned with results from previous ones [8–10] and it can be hypothesized that a few potential factors may explain our unique observations in comparison to previous studies.

First, this study used the same mechanical apparatus as Mikhail et al. [9] to apply the SMTs, which enabled SMT to be delivered using a standardized and reproducible method on all specimens. Mikhail et al. [9] delivered a total of 8 different force–time characteristics simulating 4 spinal mobilizations (MOB) and 4 SMTs to 33 asymptomatic adults and observed greater transmitted forces in 93% of SMT and 84% of MOB. Of the four unique SMT force–time characteristics used in their study, only one was similar to those used in the current study (20 N preload force, 200 N peak force, 100 ms thrust duration, 1800 N/s rate of force application). Other SMT characteristics presented a lower rate of force application (800 N/s, 32 0 N/s and 720 N/s), shorter thrust duration (100 ms) or lower peak force (100 N). Interestingly, Mikhail et al. [9] revealed an overall greater transmitted force, with increased peak force or thrust duration, but different results were observed in the current study. Specifically, lower SMT peak forces or shorter thrust durations in cadaveric specimens led to greater transmitted forces and resulted in an increased difference in force (i.e., a greater “force amplification” phenomenon). Meanwhile, a similar effect related to the rate of force application was observed in the current study and in that of Mikhail et al. [9]. Indeed, both studies revealed an increase in the “force amplification” phenomenon with the increase in the rate of force application. However, given the variations in the number of SMTs delivered and their force–time characteristics between the two studies, it is important to interpret these differences and similarities with caution. Studies comparing similar SMT force–time characteristics between living humans and cadaveric specimens are required to further clarify the role of the SMT force–time characteristics on the transmitted forces through the human thorax.

When it is not possible or ethically acceptable to carry out research on living human beings, human or animal cadaveric specimens are commonly used to study SMT biomechanics (ex. [23–26]). Despite the differences between living versus cadaveric tissue mechanical behaviours (e.g., due to water content), the use of cadaveric specimens offers the opportunity of controlling for variables that are challenging to control in living humans, such as muscle reflexes, air pressure into the lungs, and blood flow. However, the use of fresh (unembalmed and unfrozen) specimens should always be preferred over fresh-frozen or embalmed specimens due to the potential changes in the mechanical properties of viscoelastic tissue [27]. In the current study, the use of fresh cadavers was not possible due to the short time available for evaluating fresh specimens as well as limitations in laboratory space and staff availability. While conflicting results exist in the literature regarding the effect of cryopreservation on tissue mechanical properties [27–30], it is currently suggested that fresh-frozen specimens are the most suitable alternative when viscoelastic properties are being investigated [27]. Although our study findings highlight the usefulness of using cadaveric specimens when studying SMT to isolate the effect of individual characteristics on force transmission, caution should be exercised when comparing our results to those of studies evaluating living humans.

In addition to the most obvious difference between the subject receiving the SMT (cadaveric vs. living), the demographic characteristics were also considerably different, with cadaveric specimens generally presenting an older age and lower weight than participants in previous studies [8–10]. As previously discussed, only one of the eight SMTs used in Mikhail et al. [9] is similar to the ones used in the current study, namely the one with a 20 N preload force, a 200 N peak force, a 100 ms thrust duration, and a 1800 N/s rate of force application. Interestingly, Mikhail et al. [9] observed greater transmitted forces, in comparison to the applied ones, in all participants (n = 33), while this behaviour was only observed in four of the nine cadaveric specimens of the current study. Although there are slight differences in the protocols, which may partly explain the distinct results (e.g., the removal of the table cushion in the current study, but not in the Mikhail et al. [9] one), such discrepancies between the results of these studies provide additional support for our previous findings that force transmission through the thorax is not solely mediated by the SMT force–time characteristics, but also by the characteristics of the person receiving the SMT. Participants in Mikhail et al. [9] were younger (mean of 24.15 years vs. median of 82.00 years) and had thicker thorax (19.0 median cm vs. median 17.5 cm) than the specimens evaluated in the current study. Interestingly, thicker cadaveric specimens were more likely to show greater transmitted forces than applied ones, suggesting that future investigations measuring the SMT forces at the patient-table interface should normalize their force data according to the thickness of the participants’ thorax. Unfortunately, to date, this variable has only been reported by Mikhail et al. [9] and in the current study. It remains unknown if other characteristics (e.g., thorax body composition) would also need such standardization. Thorax from older adults have been observed to exhibit a unique biomechanical behaviour compared to younger adults, generally presenting lower transmitted forces than applied ones [11]. According to Funabashi et al. [11], this could potentially be related to degenerative changes, which is also likely to occur in cadaveric specimens. This provides additional confirmation that degenerative changes may influence the SMT force behaviour within the human body.

This is the first study to measure vertebral kinematics during the SMT application with simultaneous measurement of forces at both interfaces. The significant associations found with the difference in force between the interfaces might be related to the associations between vertebral displacements and SMT force–time characteristics. In the current study, increasing both peak force and thrust duration led to an increase in the relative displacement between T7 and T8. In contrast, increasing the rate of force application was associated to a decrease in the absolute displacement of T6. These results are not consistent with the current knowledge regarding the effects of SMT force–time characteristics on vertebral displacements. Indeed, absolute displacement in the vicinity of the contact area has been shown to increase with the increase in peak force and in thrust duration [19]. Relative displacement has been shown to increase with an increase in the rate of force application and a decrease in thrust duration [31]. In the current study, several kinematic variables were found to be significant when tested independently with F_diff_ and F_diff%_. Given the collinearity between kinematic variables, only the variable presenting the lowest *p* value in the invariable linear regression was included in the final model. However, other variables were also tested, and it was possible to observe that the estimate and its sign (positive or negative) were different depending on the kinematics variable. Results regarding the associations between the kinematics and the difference in force variables should therefore be interpreted with caution.

### Strength and limitation

The main strength of this study was the use of fresh cadaveric specimens, allowing the application of 25 SMTs, which would not be ethically acceptable in living humans. The use of cadaveric specimens also minimized the influence of breathing and muscle reflexes triggered by SMT observed in living humans [19] making our results less subject to confounding variables. The control of the SMT force–time characteristics by using a mechanical apparatus developed to simulate SMTs provided the opportunity of generating a standardized incremental increase in the peak force and the rate of force application. Some limitations should also be acknowledged. First, while the use of a mechanical apparatus standardized the delivery of SMT in a posterior anterior (PA) direction, it did not ensure perpendicular delivery of SMT to the thoracic kyphosis for all cadaveric specimens. The impact of this variation on the results is not fully understood and represents a limitation of the study. Considering the limited number of variables that could be included in our model due to the small sample size, the variables used in the final model were chosen on the basis of their significance when independently introduced in the level 1 based model (i.e., *p* value and marginal R^2^). On the other hand, the SMT level of our analysis was very robust, with over 200 observations. Six of the 9 specimens used in this study were females, which may potentially influence the results of this study due to sex differences related to spinal stiffness, bone density, chest geometry, and their influence on force distribution. Consequently, results from this study should be interpreted with caution. It is also important to acknowledge that a potential sequence effect of SMT application due to multiple SMTs delivered in a short period of time cannot be ruled out. While the tissues were preconditioned by the first two measurements of spinal stiffness, which were subsequently discarded from the analysis, it is possible that the observed effects in this study were influenced by changes in tissue behaviour. However, due to the risk of specimen damage with higher loads, trials were not randomized. Spinal stiffness was measured solely at the beginning of data collection; however, it would have been interesting to reassess this parameter at the end of data collection to determine whether the mechanical properties of spinal tissues were affected by multiple SMTs. One potential direction for future studies would be to compare spinal stiffness measurements before and after multiple SMTs to investigate the potential effects of SMT on the mechanical properties of spinal tissue. In addition, changes in lung air pressure during SMTs were not measured, and may have influenced the results. Although the cadavers arm position was standardized to control for potential biomechanical differences related to different arm positions, the use of hands-above-head positioning may not fully represent the variations in arm position that occur in clinical practice. Further investigation is necessary to evaluate the effects of different arm positions on force transmission during SMT. Finally, although the study used a wide range of forces typically used in a clinical setting, the way those forces were applied in the study (using a contact surface area smaller than that of a hand, and only using a posterior-to-anterior vector, regardless of spinal curvature) differs from the way that those forces would be applied in a clinical context. Because of this difference in how the forces are applied, the results of the study may not be directly applicable or generalizable to real-world clinical applications.

### Clinical implication

The basic research nature of this study limits clinical applications of the results. Clinicians should be aware that, when delivering prone thoracic SMT, the anterior portion of their patient’s thorax might sustain lower or greater force than the force they apply at their patient’s back. The factor explaining a reduction or an amplification of the applied force is not yet fully understood, but patients with thick thorax have a greater possibility to sustain a greater force in the anterior part of the thorax during prone SMT procedures. SMT peak force, thrust duration and rate of force application, as well as degenerative changes, may further modify the force transmission. Although the role of this difference in forces on the SMT clinical effects and safety requires further investigation, clinicians should consider using lower peak forces and rate for force application in patients with increased risk of anterior thorax lesion.

## Conclusion

When measured at the patient-table interface, thoracic SMT peak force can be lower or greater than the applied force at the patient’s back. Difference in force is influenced by SMT characteristics (peak force, thrust duration and rate of force application) as well as by the thoracic thickness. How the difference in force is associated with vertebral displacement in the vicinity of the contacted area remains unclear. Future studies investigating neuromechanical responses to SMT should consider assessing SMT force–time profiles at both the clinician-patient and the patient-table interfaces. Although the clinical relevance of SMT force profiling remains to be established, clinicians should consider thorax thickness as a possible modulator of forces being transmitted through the thorax during prone SMT procedures.

### Supplementary Information

**Additional file 1**. Table presenting indenter and vertebral displacements from thrust initiation to peak force for the 25 SMTs.

**Additional file 2**. Figure allowing visualization of the variation in F_diff_ (figure 1A) and F_diff%_ (figure 1B) in function of the 5 distinct SMT peak force. The results of the 5 rate of force applications for each SMT peak force are depicted per specimen.

**Additional file 3**. Figure allowing visualization of the variation in F_diff_ (figure 2A) and F_diff%_ (figure 2B) in function of the thoracic thickness measurement. Results obtained for the 25 SMTs are depicted for each specimen.

## Data Availability

Data and materials will be provided on request to the corresponding author.
