# Supplementary material for: Investigation of the factors influencing spinal manipulative therapy force transmission through the thorax: a cadaveric study
Source: Chiropr Man Therap. 2023 Aug 7;31:24. doi: 10.1186/s12998-023-00493-1 (PMC10405484; doi:10.1186/s12998-023-00493-1)
Supplement: Supplementary file 1 — Additional file 1. Table presenting indenter and vertebral displacements from thrust initiation to peak force for the 25 SMTs. [file 12998_2023_493_MOESM1_ESM.docx]

Supplementary table 1. Indenter and vertebral displacements from thrust initiation to peak force for the 25 SMTs.

|  | **Indenter displacement** | | **Axis** | **Absolute displacement (mm; mean ± SD)** | | | **Relative displacement (mean ± SD)** | |
| --- | --- | --- | --- | --- | --- | --- | --- | --- |
|  | **Total**  **(mm; mean ± SD)** | **As a percentage of chest thickness**  **(%, median ± IQR)** |  | **T6** | **T7** | **T8** | **T6/7** | **T7/8** |
| SMT1 | 10.59 ± 2.24 | 6.26 ± 1.20 | Z-axis | 6.15 ± 1.97 | 6.2 ± 1.68 | 5.61 ± 1.48 | -0.05 ± 0.59 | 0.52 ± 0.48* |
|  |  |  | Total | 6.57 ± 2.09 | 6.72 ± 1.88 | 6.20 ± 1.73 | 1.12 ± 0.46 | 0.78 ± 0.30 |
| SMT2 | 9.27 ± 1.61 | 5.48 ± 1.17 | Z-axis | 5.71 ± 1.89 | 5.75 ± 1.62 | 5.19 ± 1.47 | -0.04 ± 0.51 | 0.52 ± 0.25* |
|  |  |  | Total | 5.98 ± 1.95 | 6.12 ± 1.77 | 5.64 ± 1.69 | 1.08 ± 0.41 | 0.76 ± 0.26 |
| SMT3 | 8.84 ± 1.62 | 5.32 ± 1.18 | Z-axis | 5.50 ± 1.91 | 5.57 ± 1.67 | 5.05 ± 1.51 | -0.07 ± 0.49 | 0.47 ± 0.39* |
|  |  |  | Total | 5.74 ± 1.97 | 5.90 ± 1.81 | 5.47 ± 1.71 | 1.03 ± 0.38 | 0.70 ± 0.25 |
| SMT4 | 8.53 ± 1.62 | 5.06 ± 1.36 | Z-axis | 5.58 ± 2.02 | 5.58 ± 1.71 | 5.20 ± 1.76 | 0.00 ± 0.56 | 0.45 ± 0.53* |
|  |  |  | Total | 5.80 ± 2.07 | 5.93 ± 1.86 | 5.59 ± 1.91 | 1.07 ± 0.36 | 0.68 ± 0.21 |
| SMT5 | 8.39 ± 1.60 | 4.97 ± 1.28 | Z-axis | 5.74 ± 2.07 | 5.79 ± 1.82 | 5.29 ± 1.71 | -0.05 ± 0.45 | 0.47 ± 0.27* |
|  |  |  | Total | 5.95 ± 2.14 | 6.12 ± 1.97 | 5.71 ± 1.88 | 1.02 ± 0.41 | 0.69 ± 0.19 |
| SMT6 | 14.77 ± 2.40 | 8.49 ± 1.39 | Z-axis | 9.82 ± 2.57 | 9.75 ± 2.19 | 8.84 ± 2.10 | 0.07 ± 0.84 | 1.01 ± 0.68* |
|  |  |  | Total | 10.19 ± 2.72 | 10.39 ± 2.38 | 9.59 ± 2.25 | 1.94 ± 0.97 | 1.20 ± 0.37 |
| SMT7 | 14.22 ± 2.45 | 8.28 ± 1.39 | Z-axis | 9.29 ± 2.55 | 9.23 ± 2.18 | 8.33 ± 2.05 | 0.06 ± 0.79 | 1.00 ± 0.66* |
|  |  |  | Total | 9.64 ± 2.69 | 9.86 ± 2.40 | 9.05 ± 2.23 | 1.91 ± 1.02 | 1.19 ± 0.38 |
| SMT8 | 13.91 ± 2.47 | 8.04 ± 1.42 | Z-axis | 9.06 ± 2.53 | 8.97 ± 2.19 | 8.11 ± 2.04 | 0.08 ± 0.76 | 0.95 ± 0.46* |
|  |  |  | Total | 9.38 ± 2.66 | 9.57 ± 2.37 | 8.79 ± 2.20 | 1.88 ± 0.96 | 1.17 ± 0.37 |
| SMT9 | 13.51 ± 2.40 | 7.88 ± 1.34 | Z-axis | 8.77 ± 2.49 | 8.45 ± 2.17 | 7.86 ± 2.00 | 0.04 ± 0.72 | 0.83 ± 0.51* |
|  |  |  | Total | 9.07 ± 2.62 | 8.98 ± 2.32 | 8.53 ± 2.17 | 1.55 ± 0.55 | 1.06 ± 0.37 |
| SMT10 | 13.25 ± 2.47 | 7.86 ± 1.39 | Z-axis | 8.66 ± 2.71 | 8.59 ± 2.21 | 7.81 ± 2.07 | 0.18 ± 0.63 | 0.83 ± 0.58* |
|  |  |  | Total | 8.99 ± 2.87 | 9.15 ± 2.39 | 8.47 ± 2.24 | 1.65 ± 0.76 | 1.10 ± 0.38 |
| SMT11 | 19.71 ± 3.43 | 11.96 ± 1.70 | Z-axis | 13.68 ± 3.19 | 13.35 ± 2.77 | 12.16 ± 2.67 | 0.33 ± 0.94 | 1.23 ± 1.16* |
|  |  |  | Total | 14.11 ± 3.41 | 14.12 ± 2.96 | 13.03 ± 2.78 | 2.86 ± 1.46 | 1.56 ± 0.57 |
| SMT12 | 19.36 ± 3.34 | 11.72 ± 1.80 | Z-axis | 12.82 ± 3.04 | 12.51 ± 2.81 | 11.50 ± 2.54 | 0.10 ± 1.35 | 1.20 ± 1.08* |
|  |  |  | Total | 13.28 ± 3.27 | 13.19 ± 2.93 | 12.38 ± 2.68 | 2.42 ± 0.95 | 1.51 ± 0.69 |
| SMT13 | 18.90 ± 3.35 | 11.53 ± 1.48 | Z-axis | 12.62 ± 3.09 | 12.34 ± 2.66 | 11.23 ± 2.55 | 0.27 ± 0.99 | 1.22 ± 0.87* |
|  |  |  | Total | 13.06 ± 3.28 | 13.02 ± 2.78 | 12.07 ± 2.68 | 2.49 ± 1.08 | 1.52 ± 0.62 |
| SMT14 | 18.56 ± 3.29 | 11.40 ± 1.46 | Z-axis | 12.40 ± 3.09 | 12.18 ± 2.69 | 11.08 ± 2.60 | 0.22 ± 0.94 | 1.25 ± 0.94* |
|  |  |  | Total | 12.82 ± 3.26 | 12.84 ± 2.8 | 11.87 ± 2.71 | 2.52 ± 1.20 | 1.51 ± 0.63 |
| SMT15 | 18.30 ± 3.32 | 11.25 ± 1.48 | Z-axis | 12.10 ± 3.09 | 12.04 ± 2.73 | 10.92 ± 2.64 | 0.06 ± 1.13 | 1.23 ± 0.95* |
|  |  |  | Total | 12.51 ± 3.24 | 12.69 ± 2.84 | 11.69 ± 2.73 | 2.58 ± 1.28 | 1.55 ± 0.63 |
| SMT16 | 25.27 ± 4.59 | 14.67 ± 1.79 | Z-axis | 17.34 ± 4.42 | 15.78 ± 3.98 | 15.49 ± 4.45 | -0.12 ± 0.73 | 1.11 ± 1.32* |
|  |  |  | Total | 17.85 ± 4.71 | 16.27 ± 3.68 | 16.14 ± 4.16 | 2.55 ± 0.57 | 2.22 ± 0.52 |
| SMT17 | 24.99 ± 4.85 | 14.13 ± 2.23 | Z-axis | 16.56 ± 4.02 | 16.29 ± 3.98 | 15.25 ± 4.11 | 0.46 ± 1.17 | 1.52 ± 1.25* |
|  |  |  | Total | 17.12 ± 4.34 | 17.13 ± 4.22 | 16.24 ± 4.20 | 3.04 ± 1.29 | 2.06 ± 0.44 |
| SMT18 | 24.42 ± 4.41 | 13.98 ± 1.89 | Z-axis | 16.08 ± 3.98 | 15.32 ± 4.39 | 14.63 ± 3.78 | 0.96 ± 2.22 | 1.51 ± 1.40* |
|  |  |  | Total | 16.65 ± 4.19 | 16.16 ± 4.59 | 15.60 ± 3.83 | 3.26 ± 1.93 | 2.52 ± 1.41 |
| SMT19 | 23.86 ± 4.29 | 13.81 ± 2.45 | Z-axis | 15.56 ± 3.86 | 15.71 ± 3.79 | 14.38 ± 3.78 | -0.15 ± 1.23 | 1.52 ± 1.32* |
|  |  |  | Total | 16.17 ± 4.00 | 16.51 ± 3.82 | 15.33 ± 3.80 | 2.78 ± 1.03 | 2.06 ± 0.77 |
| SMT20 | 23.63 ± 4.29 | 13.77 ± 2.57 | Z-axis | 14.96 ± 3.88 | 15.44 ± 3.73 | 14.20 ± 3.75 | -0.03 ± 1.13 | 1.45 ± 1.05* |
|  |  |  | Total | 15.61 ± 4.09 | 16.22 ± 3.78 | 15.11 ± 3.77 | 2.43 ± 0.51 | 1.92 ± 0.60 |
| SMT21 | 29.30 ± 5.08 | 16.48 ± 1.93 | Z-axis | 20.10 ± 4.87 | 19.59 ± 4.74 | 18.59 ± 4.70 | 0.42 ± 1.55 | 1.87 ± 0.54* |
|  |  |  | Total | 20.81 ± 5.12 | 20.74 ± 5.10 | 19.83 ± 4.81 | 3.58 ± 1.29 | 2.31 ± 0.37 |
| SMT22 | 28.66 ± 5.08 | 16.04 ± 1.21 | Z-axis | 19.53 ± 4.91 | 18.94 ± 4.62 | 17.82 ± 4.41 | 0.44 ± 1.48 | 1.96 ± 0.47* |
|  |  |  | Total | 20.20 ± 5.10 | 20.05 ± 4.95 | 18.95 ± 4.55 | 3.50 ± 1.30 | 2.27 ± 0.31 |
| SMT23 | 28.21 ± 4.93 | 15.88 ± 1.35 | Z-axis | 19.03 ± 4.73 | 18.66 ± 4.64 | 17.35 ± 4.24 | 0.44 ± 1.27 | 2.03 ± 0.38* |
|  |  |  | Total | 19.68 ± 4.93 | 19.77 ± 5.00 | 18.49 ± 4.44 | 3.39 ± 1.23 | 2.29 ± 0.34 |
| SMT24 | 27.91 ± 4.90 | 15.80 ± 1.27 | Z-axis | 18.91 ± 4.85 | 18.35 ± 4.60 | 17.26 ± 4.42 | 0.40 ± 1.35 | 1.98 ± 0.39* |
|  |  |  | Total | 19.56 ± 5.02 | 19.47 ± 4.98 | 18.38 ± 4.60 | 3.42 ± 1.24 | 2.23 ± 0.35 |
| SMT25 | 26.78 ± 4.45 | 15.57 ± 1.66 | Z-axis | 18.20 ± 4.94 | 17.96 ± 4.25 | 16.27 ± 3.88 | 0.24 ± 1.30 | 1.76 ± 0.49* |
|  |  |  | Total | 18.87 ± 5.18 | 19.05 ± 4.64 | 17.50 ± 4.34 | 3.13 ± 1.39 | 2.40 ± 0.68 |

***Median and IQR are reported as they did not present a normal distribution.
