# Supplementary material for: Investigation of the factors influencing spinal manipulative therapy force transmission through the thorax: a cadaveric study
Source: Chiropr Man Therap. 2023 Aug 7;31:24. doi: 10.1186/s12998-023-00493-1 (PMC10405484; doi:10.1186/s12998-023-00493-1)
Supplement: Supplementary file 2 — Additional file 2. Figure allowing visualization of the variation in Fdiff (figure 1A) and Fdiff% (figure 1B) in function of the 5 distinct SMT peak force. The results of the 5 rate of force applications for each SMT peak force are depicted per specimen. [file 12998_2023_493_MOESM2_ESM.docx]

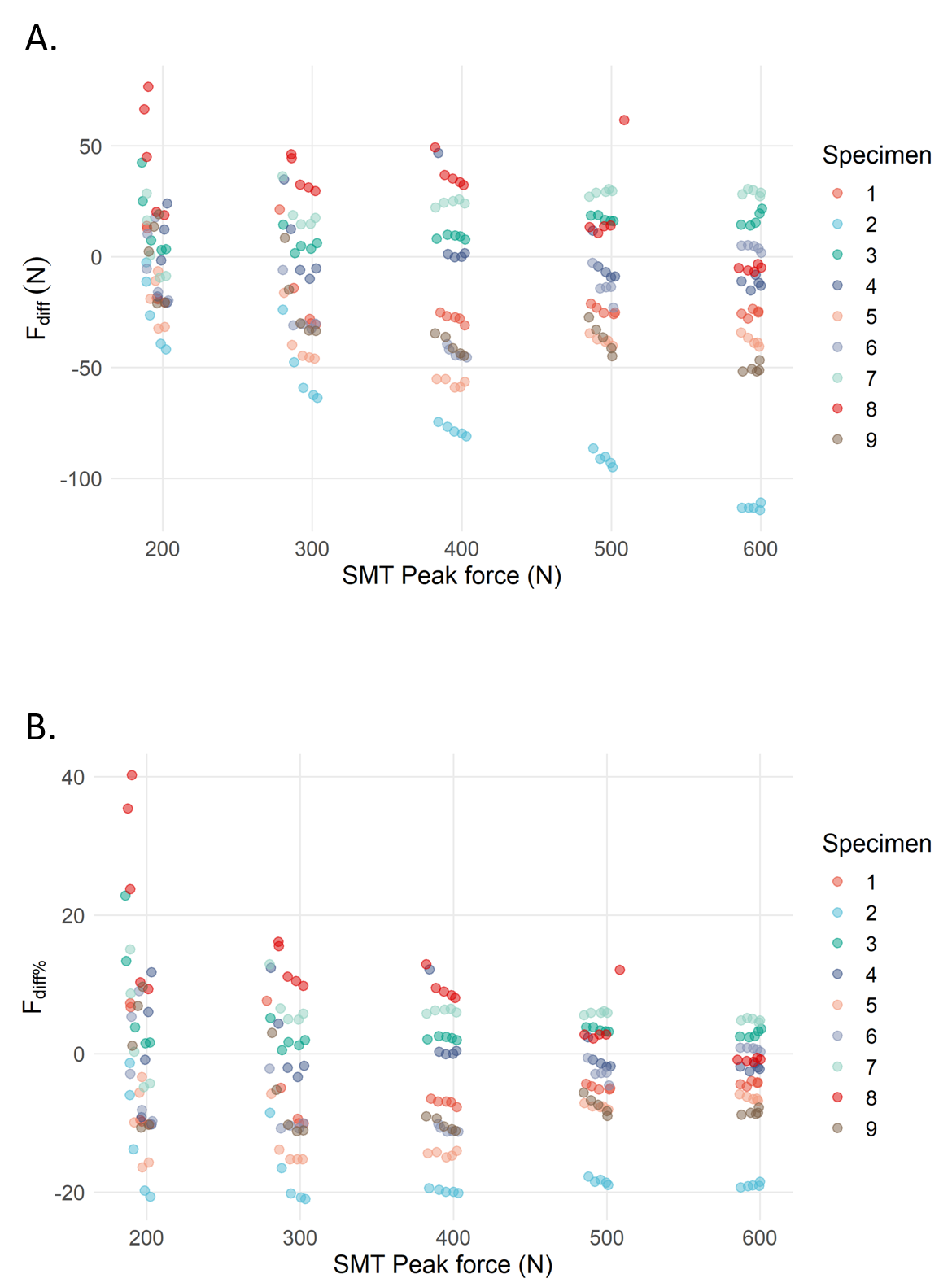


Supplementary figure 1. Visualization of the variation in F_diff_ (figure 1A) and F_diff%_ (figure 1B) in function of the 5 distinct SMT peak force. The results of the 5 rate of force applications for each SMT peak force are depicted per specimen.
