# Supplementary material for: Investigation of the factors influencing spinal manipulative therapy force transmission through the thorax: a cadaveric study
Source: Chiropr Man Therap. 2023 Aug 7;31:24. doi: 10.1186/s12998-023-00493-1 (PMC10405484; doi:10.1186/s12998-023-00493-1)
Supplement: Supplementary file 3 — Additional file 3. Figure allowing visualization of the variation in Fdiff (figure 2A) and Fdiff% (figure 2B) in function of the thoracic thickness measurement. Results obtained for the 25 SMTs are depicted for each specimen. [file 12998_2023_493_MOESM3_ESM.docx]

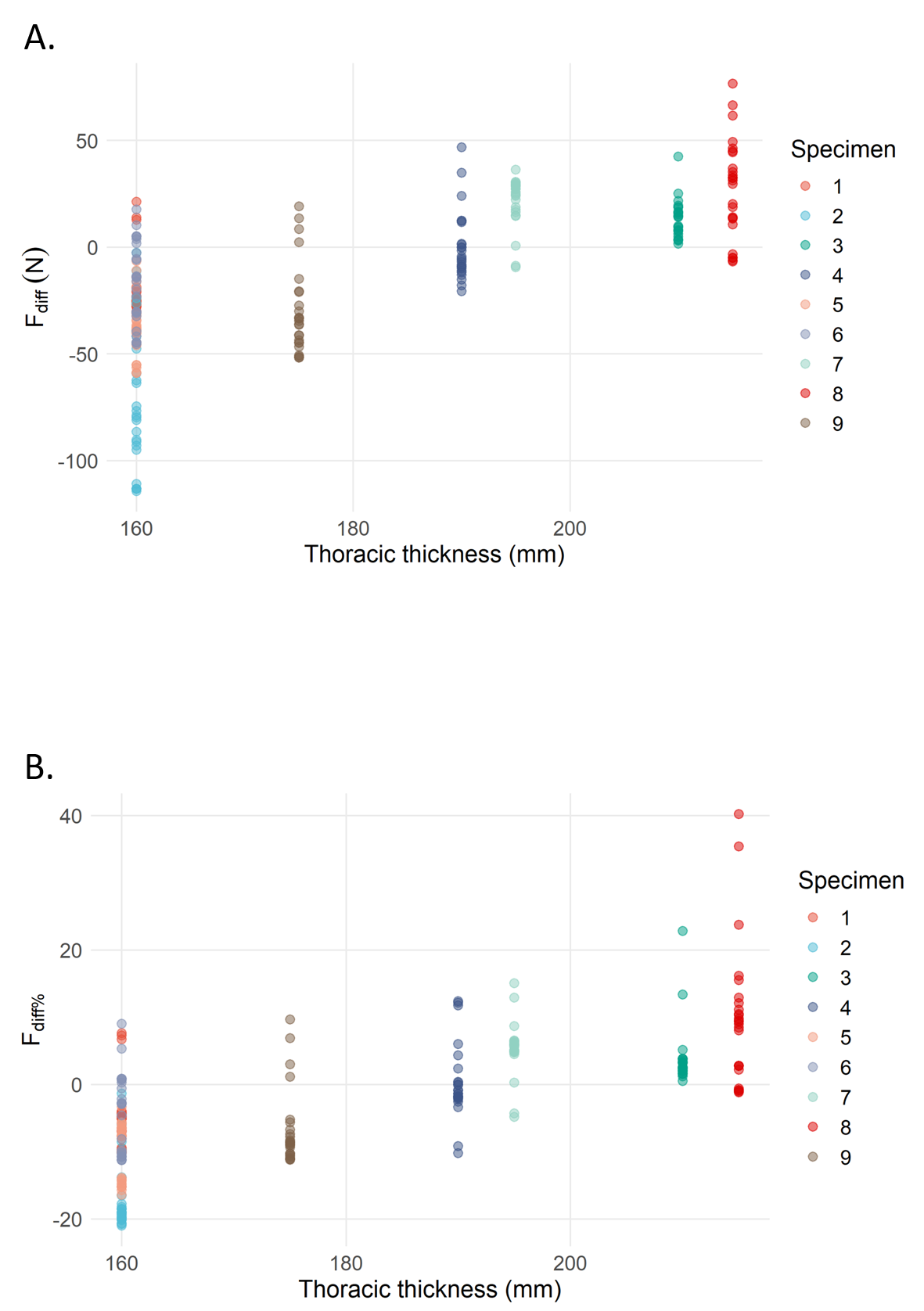


Supplementary figure 2. Visualization of the variation in F_diff_ (figure 2A) and F_diff%_ (figure 2B) in function of the thoracic thickness measurement. Results obtained for the 25 SMTs are depicted for each specimen.
